# Comparison of Different Methods for the Calculation of the Microvascular Flow Index

**DOI:** 10.1155/2012/102483

**Published:** 2012-04-23

**Authors:** Mario O. Pozo, Vanina S. Kanoore Edul, Can Ince, Arnaldo Dubin

**Affiliations:** ^1^Servicio de Terapia Intensiva, Clínica Bazterrica, Juncal 3002, C1425AYN Buenos Aires, Argentina; ^2^Cátedra de Farmacología Aplicada, Facultad de Ciencias Médicas, Universidad Nacional de La Plata, 60 y 120, 1900 La Plata, Argentina; ^3^Servicio de Terapia Intensiva, Sanatorio Otamendi y Miroli, Azcuénaga 870, C1115AAB Buenos Aires, Argentina; ^4^Department of Intensive Care Adults Erasmus MC, University Medical Centre Rotterdam, 3000 CA Rotterdam, The Netherlands

## Abstract

The microvascular flow index (MFI) is commonly used to semiquantitatively characterize the velocity of microcirculatory perfusion as absent (0), intermittent (1), sluggish (2), or normal (3). There are three approaches to compute MFI: (1) the average of the predominant flow in each of the four quadrants (MFI_by quadrants_), (2) the direct assessment during the bedside video acquisition (MFI_point of care_), and (3) the mean value of the MFIs determined in each individual vessel (MFI_vessel by vessel_). We hypothesized that the agreement between the MFIs is poor and that the MFI_vessel by vessel_ better reflects the microvascular perfusion. For this purpose, we analyzed 100 videos from septic patients. In 25 of them, red blood cell (RBC) velocity was also measured. There were wide 95% limits of agreement between MFI_by quadrants_ and MFI_point of care_ (1.46), between MFI_by quadrants_ and MFI_vessel by vessel_ (2.85), and between MFI_by point of care_ and MFI_vessel by vessel_ (2.56). The MFIs significantly correlated with the RBC velocity and with the fraction of perfused small vessels, but MFI_vessel by vessel_ showed the best *R* ^2^. Although the different methods for the calculation of MFI reflect microvascular perfusion, they are not interchangeable and MFI_vessel by vessel_ might be better.

## 1. Introduction

The patency of microvascular perfusion is essential for the preservation of aerobic metabolism and organ functions. Although the microcirculation is a key component of the cardiovascular system, its behavior may differ from that of systemic circulation [1]. Despite the continuous developments in the monitoring of critically ill patients, the evaluation of the microcirculation remained as an elusive issue during many years. The introduction of the orthogonal polarization spectral (OPS) [2] and the sidestream dark field (SDF) [3] imaging devices has recently allowed the direct visualization of microcirculation at the bedside. Thereafter, different researchers described that septic patients showed sublingual microvascular alterations such as a decreased perfusion and increased heterogeneity [3–5]. These disorders were later found to be associated with the development of multiple organ failure and death [6]. Eventually, the microcirculation became used as a therapeutic target [7–9].

Some controversies, however, still remain about the proper evaluation of the microcirculation [10]. The magnitude of the microvascular perfusion is commonly evaluated by means of the microvascular flow index (MFI) [11]. The MFI is based on determination of the predominant type of flow. For this purpose, flow is characterized as absent (0), intermittent (1), sluggish (2), or normal (3). Subsequently, the MFI has been computed in three different ways. Originally, Boerma et al. calculated the MFI as the average of the predominant flow in each of the four quadrants (MFI_by quadrants_) [11]. Then Arnold et al. reported that a determination of MFI during bedside video acquisition (MFI_point of care_) gave a good agreement with the MFI_by quadrants_ [12]. Finally, Dubin et al. used the mean value of the MFI determined in each individual vessel (MFI_vessel by vessel_) [1, 8, 9]. This analysis is time consuming but tightly correlated with the actual red blood cell (RBC) velocity measured with a software both in experimental and clinical conditions [1, 13, 14].

Our hypothesis was that the agreement between the different methods to determine the MFI is poor and that the MFI_vessel by vessel_ better reflects the microvascular perfusion than the other approaches.

## 2. Materials and Methods

This was a prospective observational study performed in a teaching intensive care unit. It was approved by the Institutional Review Board. Informed consent was obtained from the next of kin for all patients admitted to the study.

One hundred videos were obtained by a single operator (AD) from 25 patients with septic shock in different clinical and hemodynamic conditions. Their clinical and epidemiologic characteristics are shown in Table 1. All the patients were mechanically ventilated and received infusions of midazolam and fentanyl. Corticosteroids, propofol, and activated protein C were never used.

The microcirculatory network was evaluated in the sublingual mucosa by means of a SDF imaging device (Microscan, MicroVision Medical, Amsterdam, Netherlands) [3]. Different precautions were taken and steps followed to obtain images of adequate quality and to ensure good reproducibility. Video acquisition and image analyses were performed by well-trained researchers. After gentle removal of saliva by isotonic-saline-drenched gauze, steady images of at least 20 seconds were obtained while avoiding pressure artifacts through the use of a portable computer and an analog/digital video converter (ADVC110, Canopus Co, San Jose, CA, USA). Videoclips were stored as AVI files to allow computerized frame-by-frame image analysis. Adequate focus and contrast adjustment were verified and images of poor quality discarded. The entire sequence was used to characterize the semiquantitative characteristics of microvascular flow and particularly the presence of stopped or intermittent flow.

MFI was randomly and blindly determined in three different ways by a single researcher (MOP). First, a semiquantitative analysis by eye was performed in individual vessels. It distinguishes between no flow (0), intermittent flow (1), sluggish flow (2), and continuous flow (3) [11]. A value was assigned to each individual vessel. The overall score of each video is the average of the individual values (MFI_vessel by vessel_). In addition, MFI_by quadrants_ was calculated as the mean value of the predominant type of flow in each of the four quadrants. Finally, as an approximation to the real-time assessment at the bedside [12], MFI_point of care_ was determined during a 20-second observation of a video sequence.

We also calculated the proportion of perfused small vessels as the number of vessels with flow values of 2 and 3 divided by the total number of vessels.

Quantitative RBC velocity of single vessels was measured through the use of space-time diagrams, which were generated by means of analysis software developed for the SDF video images [15]. This method of velocity determination consists of making diagrams of changes in grey-level values (e.g., flowing red blood cells) along the center line of a vessel segment being analyzed, as a function of time. In sequential images, the diagram of such an analysis consists of the *y*-axis, the distance traveled along the vessel segment and on the *x*-axis, time. This portrayal of the kinetics of sequential images generates slanted dark lines representing the movement of the red blood cells, the slopes of which give red blood cell velocity. This value is calculated as *v* = Δ*s*/Δ*t*, where Δ*s* is the longitudinal displacement along the vessel centerline in time fragment Δ*t*. We traced three center lines manually in the space-time diagram, and the average orientation was used to calculate the RBC velocity. The RBC velocity of each video was the average of all RBC velocities measured in single vessels in that video. The analysis was restricted to small vessels (i.e., vessels with a diameter <20 *μ*m). 

### 2.1. Statistical Analysis

The agreement between the three methods for the determination of MFI was tested using the Bland-Altman method [16]. In addition, linear regression analysis was performed between MFIs and the fraction of perfused small vessels and between MFIs and RBC velocity.

## 3. Results

For the determination of MFI_vessel by vessel_, 37 ± 9 small vessels per video were assessed. For the calculation of MFI_by quadrants_, the four quadrants were analyzed in all videos. The red blood cell velocity was measured in 20 ± 8 small vessels per video.

**Figure 1:**
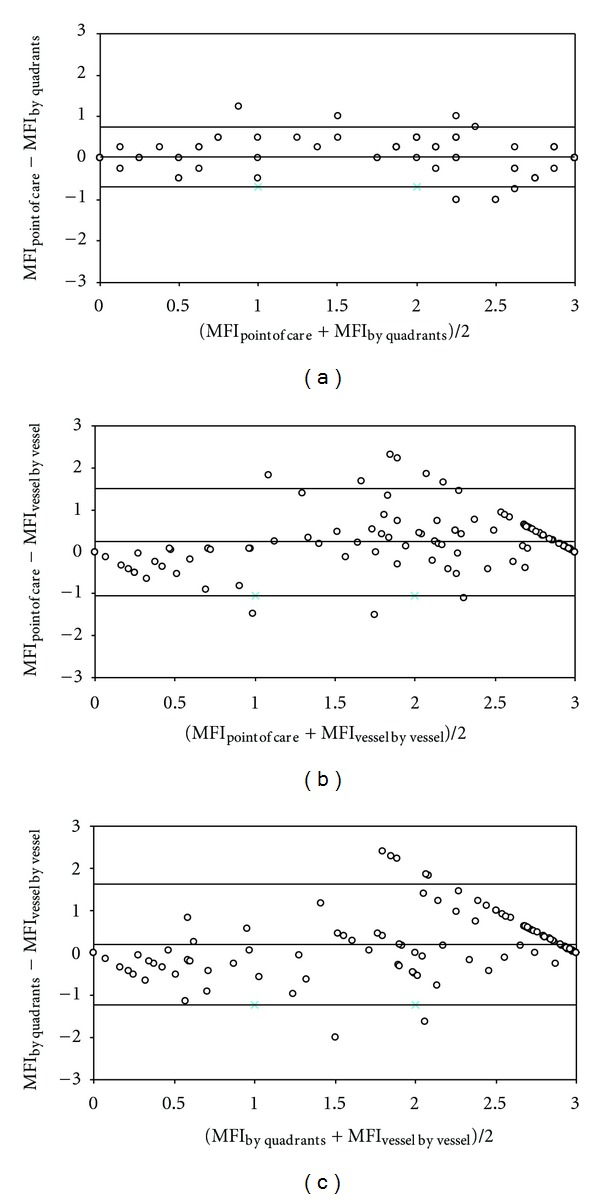
Bland and Altman analysis for the different methods used for the calculation of microvascular flow index (MFI). Panel (a): bedside point of care MFI (MFI_point of care_) and MFI determined by quadrants (MFI_by quadrants_). Panel (b): MFI_point of care_) and MFI determined by vessel by vessel analysis (MFI_vessel by vessel_). Panel (c): (MFI_by quadrants_) and (MFI_vessel by vessel_). Lines are bias and 95% limits of agreement.

Figure 1 shows the wide 95% limits of agreement among the different methods for determining MFI. The bias ± precision for MFI_point of care_ and MFI_by quadrants_ (0.03 ± 0.37) was lower than for the MFI_point of care_ and MFI_vessel by vessel_ (0.24 ± 0.65, *P* = 0.005) or MFI_by quadrants_ and MFI_vessel by vessel_ (0.21 ± 0.73, *P* = 0.05) comparisons.

**Figure 2:**
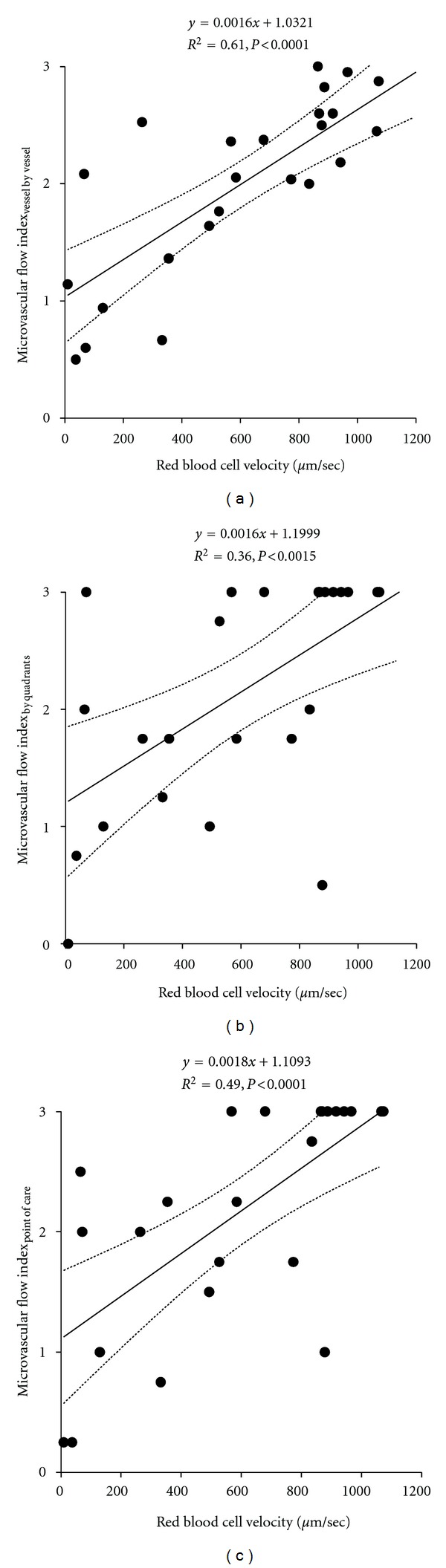
Correlations of the red blood cell velocity with the microvascular flow index determined by vessel by vessel analysis (MFI_vessel by vessel_) Panel (a), the microvascular flow index determined by quadrants (MFI_by quadrants_) Panel (b), and the bedside point-of-care microvascular flow index (MFI_point of care_) Panel (c).

 RBC velocity significantly correlated with the three MFIs (Figure 2). Although, the MFI_vessel by vessel_ method showed the highest *R* ^2^, the difference did not reach statistical significance.

**Figure 3:**
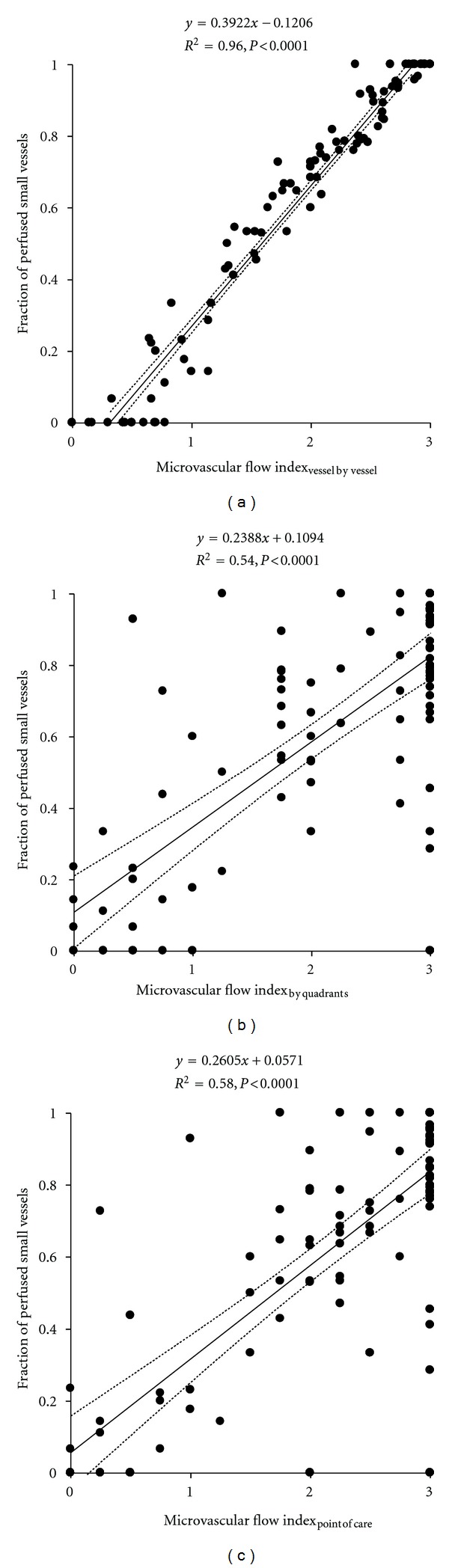
Correlations of the proportion of perfused small vessels with the microvascular flow index determined by vessel by vessel analysis (MFI_vessel by vessel_) Panel (a), the microvascular flow index determined by quadrants (MFI_by quadrants_) Panel (b), and the bedside point-of-care microvascular flow index (MFI_point of care_) Panel (c).

 The proportion of perfused small vessels exhibited significant correlations with the three methods used in the calculation of MFI (Figure 3). The MFI_vessel by vessel_ showed the highest coefficient of determination, whose value was statistically higher than the other two (*P* < 0.0001 for both).

## 4. Discussion

Our results showed that each method used for the calculation of MFI was significantly correlated with the actual RBC velocity. Nevertheless, the agreement among the different MFIs was poor. The MFI_vessel by vessel_ was the approach that had the best correlations with the RBC velocity and the proportion of perfused small vessels.

According to a recent consensus conference, the evaluation of the microcirculation should take into account the three different characteristics of density, perfusion, and flow heterogeneity. The question of which parameters are more appropriate to evaluate microcirculatory perfusion and density is still controversial. In particular, the discussion has mainly been focussed on the advantages versus the limitations of either the proportion of perfused vessels or the MFI [10]. Since the proportion of perfused vessels only distinguishes continuous from intermittent/stopped flow, the presence of a continuous but slow flow could be missed. The MFI does not provide information about functional density. Theoretically this index could be misleading if flow improves in perfused vessels, but the total number of perfused vessels also decreases. Moreover, the MFI is a categorical variable, so a change from 0 to 1 may have a different meaning in terms of tissue perfusion than a change from 2 to 3. Beyond these considerations, we found strong correlations between the proportion of perfused small vessels and the different approaches to MFI. The correlation with MFI_vessel by vessel_, however, was the strongest and also exhibited very narrow 95% confidence intervals. These findings suggest a similar performance of both the proportion of perfused small vessels and MFI in the characterization of microcirculatory perfusion, especially when the MFI_vessel by vessel_ is used.

We found statistically significant correlations between RBC velocity and the three measurements of MFI. Although the correlation with MFI_vessel by vessel_ showed the best coefficient of determination, the difference between that *r* ^2^ value and the other two did not reach statistical significance. Probably, our study was underpowered for showing this difference.

The agreement between the different approaches to the MFI was poor. We found large 95% limits of agreements between them, whose range precludes any interchangeability. The 95% limits of agreement between MFI_point of care_ and MFI_by quadrants_ were lower than those found between the other MFIs, although still wide. Arnold et al. reported a similar bias ± precision for this Bland and Altman analysis (−0.031 ± 0.198). Nevertheless, they concluded that the agreement was good.

We found positive biases with MFI_point of care_ versus MFI_vessel by vessel_ and with MFI_by quadrants_ versus MFI_vessel by vessel_, meaning that MFI_point of care_ and MFI_by quadrants_ overestimate MFI_vessel by vessel_. These biases could be anticipated since the two first methods use the predominant type of flow, either in the whole videomicroscopic area or in the quadrants. Accordingly, a high but not predominant proportion of small vessels with stopped or intermittent flow could be left unconsidered in the MFI_point of care_ and MFI_by quadrants_. In contrast, in the MFI_vessel by vessel_, every vessel score is used in the final computation. For example, if 30% of the small vessels have stopped flow and 70% normal blood flow, the MFI_vessel by vessel_ will be 2.1, while with the other two methods the predominant flow will be 3.

Although the methods are not interchangeable and MFI_vessel by vessel_ probably better reflects the velocity of the perfusion, MFI_by quadrants_ and MFI_point of care_ were also significatively correlated with the proportion of perfused vessels and the RBC velocity.

This study has certain limitations. First, the MFI_point of care_ used in this study was only a simulation of that used in the study of Arnold et al. [12]. We performed the MFI_point of care_ during a 20 sec view of the video sequence but not during a real video acquisition. In addition, the strong correlation between MFI_vessel by vessel_ and the proportion of perfused vessels could be partially explained by mathematical coupling. This problem can develop when two parameters, calculated from a shared variable, are subsequently correlated. If there is an error in the determination of the shared variable, it could be propagated in the calculation of those parameters. The resulting correlation could not be a real phenomenon but could be the expression of the methodological mistake. Mathematical coupling, however, is only applicable to artifactual relationships when there is a significant error in the measurement of the common variable. Another limitation is that the number of analyzed videos, especially those in which the RBC velocity was measured, was limited. Finally, we correlated the MFIs with other parameters of perfusion such as the proportion of perfused vessels and the RBC velocity but not with an actual measurement of microvascular flow.

In conclusion, although the different methods for the calculations of MFI reflect the magnitude of microvascular perfusion, they are not interchangeable. Even though the MFI_vessel by vessel_ is time consuming, this method could arguably more precisely track the microcirculatory perfusion as suggested by its stronger correlations with other parameters of microvascular perfusion. Larger studies are needed to determine if these findings also imply advantages as an outcome predictor.

## Figures and Tables

**Table 1:** Clinical and epidemiologic characteristics of the patients.

Age, years	73 ± 10
Gender male, *n* (%)	14 (56)
SOFA score	10 ± 3
APACHE II score	25 ± 6
Actual mortality, %	
ICU mortality	48
30-day mortality	48
Hospital mortality	48
APACHE II predicted mortality, %	49 ± 20
Norepinephrine (*μ*g/kg/min)	0.51 ± 0.41
Intra-abdominal	8 (32)
Respiratory	8 (32)
Urinary	6 (24)
Intravascular	3 (12)

Definition of abbreviations: SOFA, sepsis-related organ failure assessment; APACHE, acute physiology and chronic health evaluation.

Data are expressed as mean ± standard deviation or number (percentage).
